# Professor Spencer F. Baird

**Published:** 1887-10

**Authors:** 


### PROFESSOR SPENCER F. BAIRD.

Professor S. F. Baird, of the United States Fish Commission, died at Woods Holl, Massachusetts, on August 19th, 1887.

Spencer Fullerton Baird was born of Scotch-German ancestry in Reading, February 3d, 1835. At the age of 17 he was graduated from Dickinson College, and his time for several years thereafter was devoted to studies in general natural history, to long pedestrian excursions for the purpose of observing and collecting specimens, and to the organization of a private cabinet of natural history, which, a few years later, became the nucleus of the museum of the Smithsonian Institution. He studied medicine and attended a course of lectures at the College of Physicians and Surgeons in New York, in 1842, but never formally completed his course, although he received the degree of M. D., Honoris Causa, from the Philadelphia Medical College in 1848.

In 1845 he was chosen professor of natural history in Dickinson College, and was subsequently elected to the chair of natural history and chemistry in the same institution. In 1850 he was made assistant secretary of the Smithsonian Institute; at the age of 27 entered upon his life work in connection with that foundation. In 1871 he was appointed by President Grant to the position of United States Fish Commissioner, an unsalaried office, and in 1878, upon the death of Professor Henry, he was, by the unanimous vote of the Regents, elected secretary of the Smithsonian Institution.

Professor Baird received the degree of physical science from Dickinson College, and that of doctor of laws from Columbian University. He received several foreign decorations. He held honorary and corresponding memberships in many of the most renowned scientific societies in the world and active membership in most of those in America.

